# Expanding the Potential of Identical Location Scanning Transmission Electron Microscopy for Gas Evolving Reactions: Stability of Rhenium Molybdenum Disulfide Nanocatalysts for Hydrogen Evolution Reaction

**DOI:** 10.1021/acsami.3c09188

**Published:** 2023-09-29

**Authors:** Miquel Vega-Paredes, Christina Scheu, Raquel Aymerich-Armengol

**Affiliations:** Max-Planck-Institut für Eisenforschung GmbH, Max-Planck-Strasse 1, Düsseldorf 40237, Germany

**Keywords:** identical location scanning transmission electron microscopy, hydrogen evolution reaction, molybdenum disulfide, electrocatalysis, stability

## Abstract

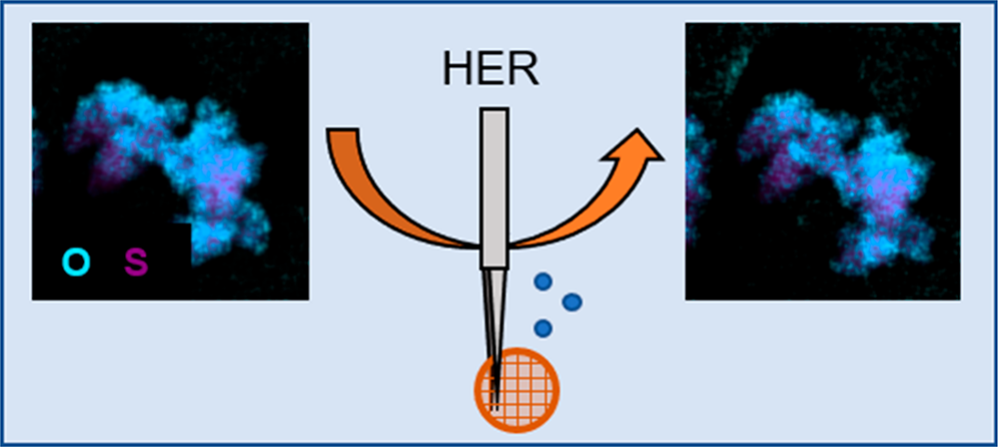

Identical location (scanning) transmission electron microscopy provides valuable insights into the mechanisms of the activity and degradation of nanocatalysts during electrochemical reactions. However, the technique suffers from limitations that hinder its widespread use for nanocatalysts of gas evolving reactions, e.g., the hydrogen evolution reaction (HER). The main issue is the production of bubbles that cause the loss of electric contact in identical location measurements, which is critical for the correct cycling of the nanocatalysts and interpretation of the electron microscopy results. Herein, we systematically evaluate different set-ups, materials, and tools to allow the facile and reliable study of the stability of HER nanocatalysts. The optimized conditions are applied for the study of layered rhenium molybdenum disulfide (Re_0.2_Mo_0.8_S_2_) nanocatalysts, a relevant alternative to Pt catalysts for the HER. With our approach, we demonstrate that although the morphology of the Re_0.2_Mo_0.8_S_2_ catalyst is maintained during HER, chemical composition changes could be correlated to the electrochemical reaction. This study expands the potential of the IL(S)TEM technique for the construction of structure–property relationships of nanocatalysts of gas evolving reactions.

## Introduction

Identical location (scanning) transmission electron microscopy (IL(S)TEM) is a powerful technique to study the stability of nanocatalysts during electrochemical reactions.^1,2^ In IL(S)TEM, the same region of a TEM specimen is analyzed before and after electrochemical testing. This methodology allows for direct correlation of the morphological and compositional changes of nanocatalysts to the electrochemical conditions they were subjected to, thus providing insights to the corrosion mechanisms^3,4^ or the nature of the active species^5^ down to the atomic scale. When compared to in situ liquid cell (S)TEM,^6^ IL(S)TEM possesses the advantages of higher spatial resolution, longer term studies of up to several thousands of potential cycles, and reduced electron beam-induced effects, which can produce undesirable side reactions^7,8^

First introduced by Mayrhofer et al.,^9,10^ IL(S)TEM was originally developed for the study of the degradation of fuel cell nanocatalysts, with several studies focusing on the effects of oxygen reduction reaction^11,12^ and ramping up/down conditions^3,13^ on the nanocatalysts. When applied to such fuel cell nanomaterials, processes like Ostwald ripening, particle detachment, movement, agglomeration, and dissolution were identified as corrosion mechanisms.^14−16^

However, beyond fuel cell technology, the IL(S)TEM method has only mildly expanded to other electrochemical application fields, such as batteries^17,18^ and water-splitting catalysis. Water-splitting converts water to oxygen and hydrogen gases through the oxygen evolution (OER) and the hydrogen evolution reaction (HER), respectively.^19−22^ These reactions have recently attracted a lot of interest as a method of obtaining green hydrogen fuel, key for the successful decarbonization of the economy.^23,24^ Nevertheless, to date only few degradation studies on materials such as Ir-based,^25−27^ perovskite^5^ or Ni/Fe-based^28^ OER catalysts have been conducted with IL(S)TEM. When it comes to HER, even fewer investigations on Pt-based nanoparticles have been reported.^29,30^

The reduced number of IL(S)TEM research on gas evolving reactions can be related to their intrinsic difficulty:^8,29^ the bubbles formed on the TEM grids do not easily release, thus blocking their surface and preventing electrical contact with the electrolyte. When the electrical contact is lost, the nanomaterial can no longer catalyze the reaction, and therefore, no corrosion mechanisms nor active species can be identified. This problem is especially severe for HER, and existing studies for gas evolving reactions had to resort to the use of expensive modified rotating disk electrodes,^30^ specialized equipment such as the modified floating electrode,^31,32^ or keeping an unrealistically low overpotential of 100 mV to avoid energic bubbling.^29^ Besides limiting the potential applications of IL(S)TEM, such pitfalls also jeopardize the correct interpretation of the results of IL(S)TEM conducted in catalysts with high electrochemical stability, e.g. noble metals or materials such as MoS_2_-based nanocatalysts,^33^ where the absence of structural corrosion could be attributed both to loss of contact due to bubbles and to the inherent stability of the material. Therefore, it is still necessary to develop a reliable and widely accessible methodology for performing IL(S)TEM on HER catalysts.

Herein, we explore different IL(S)TEM methods to enable the study of HER nanocatalysts. From different electrical connections of the TEM grid to the working electrode and materials used for the setup to the electrochemical conditions, we determine an easy approach for conducting reliable IL(S)TEM investigation for HER that can be widely implemented due to its simplicity. Moreover, we applied the optimized procedure to investigate the morphological and chemical stability of the Re_0.2_Mo_0.8_S_2_ nanocatalyst across electrochemical testing through 4000 cyclic voltammetries conducted in the range from 0 to −0.25 V_RHE_ (reference hydrogen electrode). We selected Re_*x*_Mo_1–*x*_S_2_ nanocatalysts as an ideal case study due to their high activity and stability under HER conditions,^34−100^ making the correct interpretation of the IL(S)TEM results especially critical. We found that connecting the grid to the working electrode using tweezers of an inert metal (as opposed to the typically used glassy carbon electrode) is a reliable, reproducible, and simple to implement method that can be widely adapted by the community, expanding the huge potential of IL(S)TEM to the water splitting field, where the stability of the catalysts is yet the bottleneck for the practical application of acid electrolyzers.

## Results and Discussion

### Technique Development

**Figure 1:**
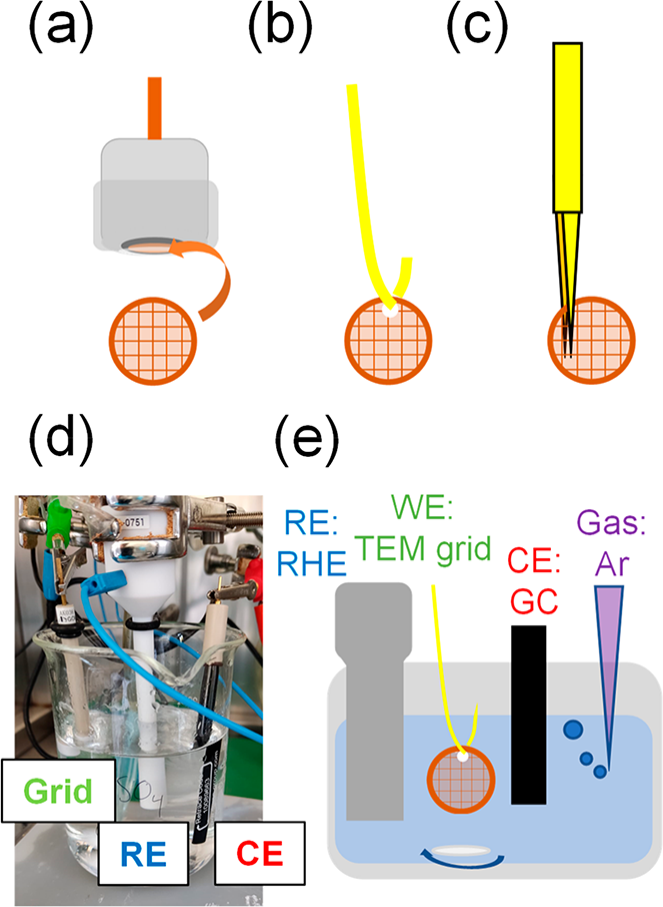
Scheme of the methods for connecting a TEM finder grid as a working electrode: (a) glassy carbon rod and a Teflon cap, (b) a conducting wire, and (c) tweezers. (d) Experimental and (e) schematic setup used for the IL experiments. RE is the reference electrode, CE is the counter electrode, and WE is the working electrode.

To enable electrochemistry on a TEM finder grid, it has to be connected as the working electrode. The electrical contact is usually ensured by fixing the grid on a glassy carbon rod electrode with a Teflon cap with a hole to ensure electrolyte-grid contact^3^ (Figure 1a). Alternatively, the TEM grid can also be connected with through a wire^36^ or tweezers^37^ of an inert material in the conditions of study (Figure 1b, c).

**Figure 2:**
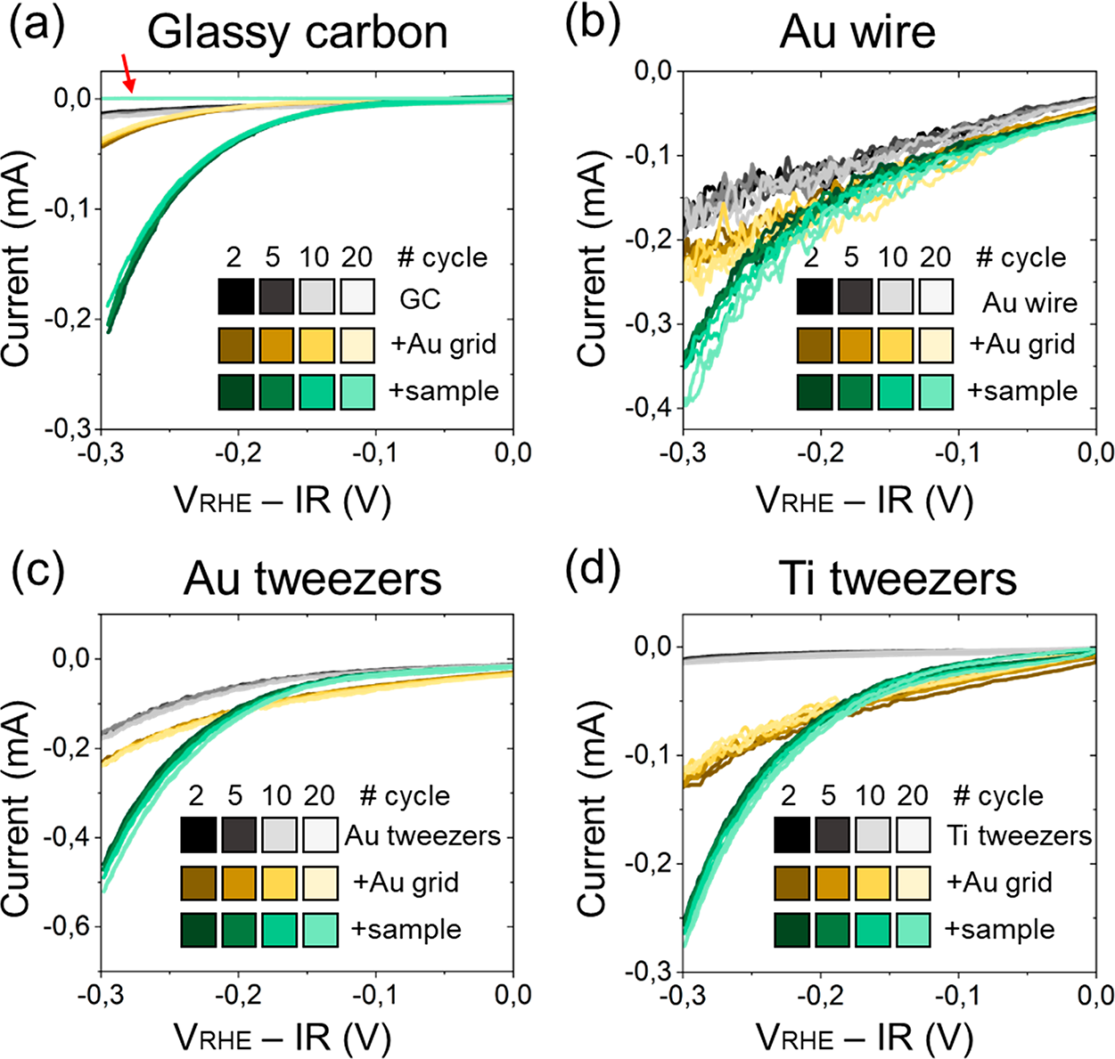
Comparison of the different methods of electrical contact of the TEM grids for IL(S)TEM, (a) glassy carbon, (b) Au wire, (c) Au tweezers, and (d) Ti tweezers. The potential was cycled from 0 to −0.3 V_RHE_ at a scan rate of 10 mV/s.

To systematically compare the performance of these methods for the study of HER, 20 cyclic voltammetries (CV) were performed with Au/C grids containing Re_0.2_Mo_0.8_S_2_ nanocatalysts at 10 mV/s of scan rate from 0 to −0.3 V_RHE_ (Figure 2). To prevent current loss derived from particle detachment, the grid was loaded with an ink of the active Re_0.2_Mo_0.8_S_2_ nanomaterial and Nafion. As blank measurements, CVs with an unloaded Au grid and without a grid were also acquired for each method.

The electrochemical results when using the glassy carbon rod electrode in Figure 2a show that the bare glassy carbon rod has a low current intensity in the potential range analyzed, which is increased when attaching an Au TEM grid confirming the electrical contact. Loading the TEM grid with the Re_0.2_Mo_0.8_S_2_ nanocatalyst resulted in larger HER current. However, the electrical contact is reduced until being completely lost during the HER, as evidenced by the current drop even below the GC baseline in the 20th CV (Figure 2a, red arrow). Specifically, the drop in current occurred at the 16th CV (Figure S1). The loss of contact is explained by the evolution of hydrogen gas from the Re_0.2_Mo_0.8_S_2_ nanocatalyst on the TEM grid. Unable to completely detach from the interface between the grid and the Teflon cap, the hydrogen bubbles grow, eventually completely covering the hole of the Teflon cap that provides contact of the electrolyte with the glassy carbon and TEM grid (see schematic in Figure S2). The experiment was also performed with a higher scan rate of 100 mV/s to account for milder gas evolution conditions, yet the contact was also lost by the 40th CV (Figure S3). Thus, the typical IL(S)TEM approach using glassy carbon rod electrodes proved to be unsuitable for the study of HER catalysts.

The use of a metallic wire as a method of electric contact (Figure 1b), in which the grid is pierced with the wire to mount it, was tested next using a Au wire. Despite minimizing the area of gold exposed from the wire by covering part of the surface with Teflon tape, the baseline current derived from the Au wire had a higher intensity (Figure 2b). This is detrimental for the IL(S)TEM measurements, as the current passing through the sample becomes very sensitive to the area of exposed Au from the wire, affecting both the stability of the nanocatalysts and the reproducibility of the measurement (Figure S4a). Nevertheless, the contact with the TEM grid with and without Re_0.2_Mo_0.8_S_2_ nanocatalyst was still confirmed in the form of a current increase. During the 20 CVs of the test, no loss of electric contact was observed, which can be attributed to the comparatively larger area of the TEM grid exposed to the electrolyte promoting bubble release to the electrolyte. However, this method of electrical contact produced noisy electrochemical data due to the movement of the gold wire and the grid itself in the stirred electrolyte as well as the stronger bubbling of hydrogen from the gold wire surface. Such bubbles were observed along the wire and distributed all across the grid surface, but especially on the interface of the Au wire and TEM grid (see schematic in Figure S5). The uncontrolled buildup of bubbles across the TEM specimen surface prevents the correct interpretation of the IL(S)TEM data, since there is no guarantee of electrical contact with the electrolyte in the areas of analysis during the electrochemical experiment. Similar results in terms of bubble build-up and data noisiness were obtained despite the attempt to lower the background current with a wire material with lower current such as W (Figure S6a). Using a Ti wire also produced data noisiness (Figure S6b). Due to these issues and the fact that the wire method of contact also compromises the structural integrity of the TEM grid as a hole needs to be made on it, an alternative method was pursued.

Finally, metallic tweezers were used as the electric contact for the TEM specimen. Figure 2c and Figure 2d show the CV results using Au and Ti tweezers, respectively. Compared to the metallic wire method, the electrochemical data acquired with tweezers are less noisy, which can be attributed to the improved fixation of the grid from one side by the tweezers. Furthermore, the bubbles are also easily detached from the surface of the grid due to the mild movement that the fixation still allows, and only a few of them stuck exclusively at the interface between the tweezers and the grid (Figure S7). Thus, (S)TEM can be conducted and interpreted as long as the areas of analysis are taken on the opposite side of the grid. Since the Au tweezers showed less reproducibility on the current response among replicates than Ti tweezers, (Figure S4b, c), the latter was the method of choice for reliable IL(S)TEM for HER. Similar results were found when increasing the scan rate to 100 mV/s (Figure S8).

### IL(S)TEM on Re_0.2_Mo_0.8_S_2_ Nanocatalysts

**Figure 3:**
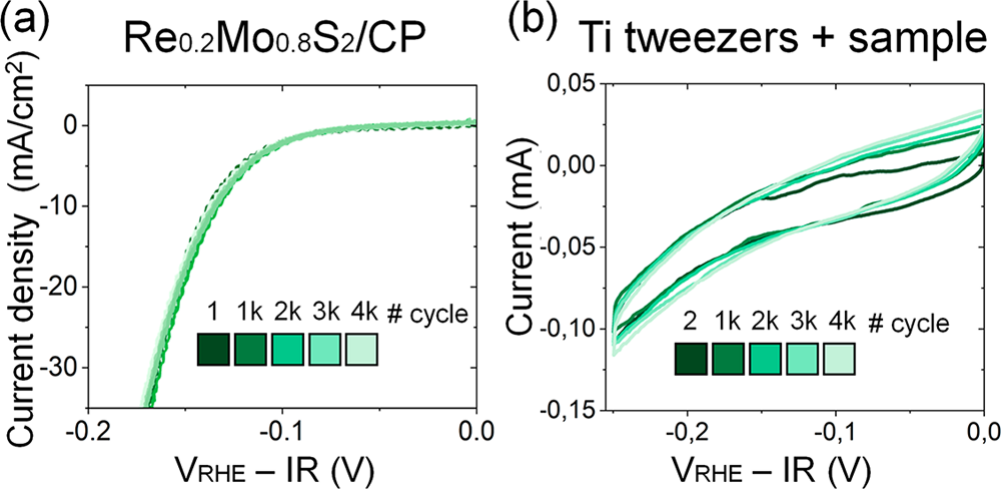
4000 CVs of Re_0.2_Mo_0.8_S_2_ acquired from 0 to −0.25 V_RHE_ (a) Re_0.2_Mo_0.8_S_2_/CP electrode. (b) Re_0.2_Mo_0.8_S_2_/Au/C TEM grid, acquired with Ti tweezers.

Before conducting ILSTEM measurements, 4000 CVs were conducted from 0 to −0.25 V_RHE_ on a Re_0.2_Mo_0.8_S_2_ electrode grown on a carbon paper substrate (Re_0.2_Mo_0.8_S_2_/CP) (Figure 3a, Figure S9). The results demonstrate that the reaction overpotential did not suffer any detectable changes, which showcases the high electrochemical stability retained by this material. To understand whether the morphology was equally maintained stable or whether some changes and/or degradation occurred, the 4000 CVs were subsequently performed in an IL(S)TEM setup using the Ti tweezers to establish the electric contact (Figure 3b). The current was maintained fairly stable across the 4000 CVs measured, with a small increase possibly derived from an activation of the Ti tweezers (Figure S10). Furthermore, the only bubbles observed after the 4000 CVs on the setup were exclusively localized on the grid-tweezer interface, far from the areas where the STEM analyses were conducted (Figure S7), which is key for a reliable interpretation of the results.

**Figure 4:**
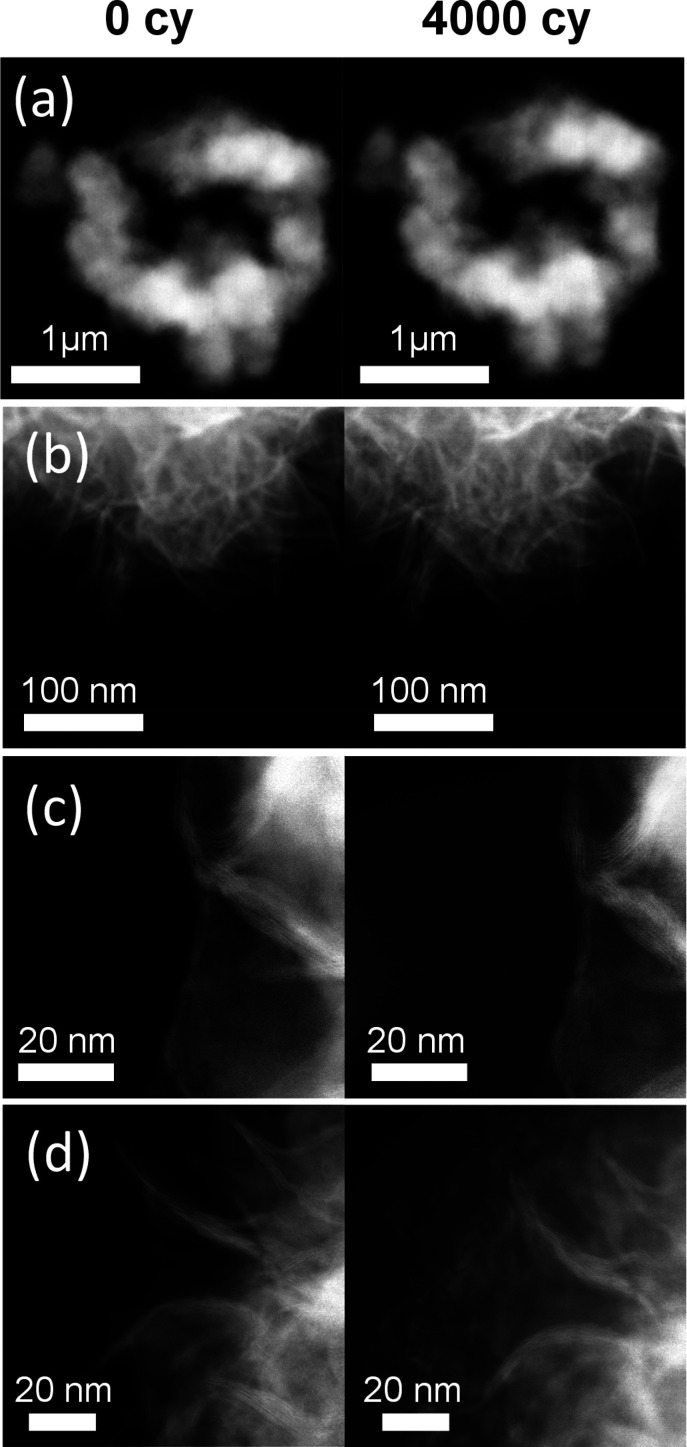
(a–-d) Different areas imaged in identical location conditions before and after 4000 CVs showing the morphology evolution of the Re_0.2_Mo_0.8_S_2_ nanocatalyst.

Figure 4 shows the morphological evolution of the Re_0.2_Mo_0.8_S_2_ nanomaterials during electrocatalysis. These catalysts possess a nanoflower structure made up from few-layered nanosheets assembled in a porous 3D structure. Such morphology is maintained down to the nanometric scale without visible changes after 4000 CVs. These results are consistent with the high stability observed in the electrochemical measurements (Figure 3). However, in a few areas at the edges of the nanoflowers, some redistributed material was observed (Figure 4d) as a result of electrochemical cycling. Energy dispersive X-ray (EDS) measurements were performed to such areas to understand the composition of the appeared layer of material. The measurements (Figure S11) revealed that the redistributed material on the edges of Re_0.2_Mo_0.8_S_2_ nanoflowers is exclusively composed of carbon, oxygen and fluorine, matching the composition of the Nafion binder used for the Re_0.2_Mo_0.8_S_2_ ink drop cast on the TEM specimen. This partial redistribution of Nafion did not hinder the morphology comparison among cycles and further confirmed a successful electrochemical cycling conducted on the grid. A similar Nafion degradation after cycling was observed in an area not previously imaged, ruling out a possible electron beam induced effect.

**Figure 5:**
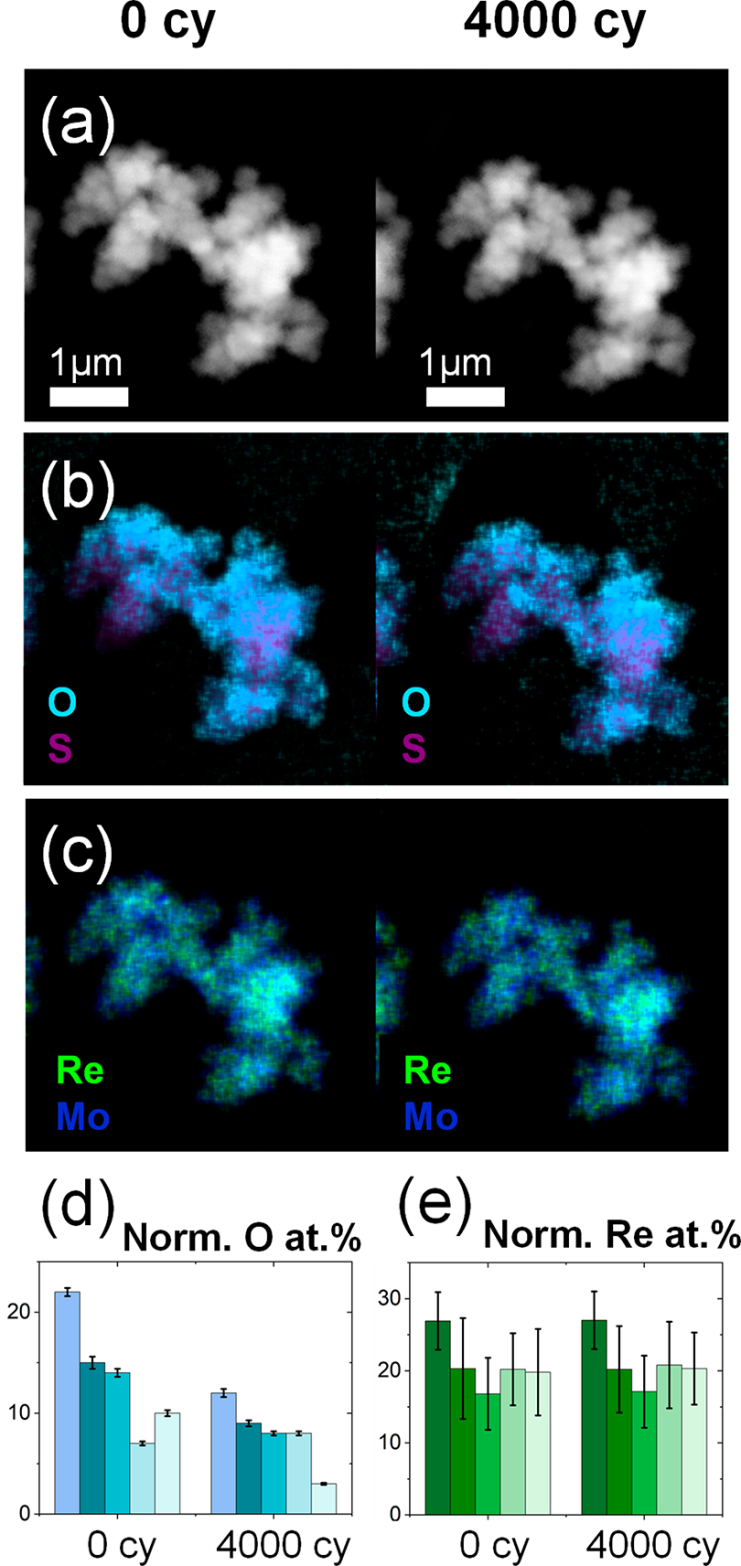
Composition changes during IL(S)TEM analyzed by EDS. (a) HAADF-STEM image and corresponding composition maps showing the distribution of (b) oxygen and sulfur and (c) rhenium and molybdenum before and after 4000 CVs. (d) Evolution of the normalized oxygen content during 4000 CV in different IL(S)TEM areas. (e) Evolution of the normalized rhenium content during 4000 CV in different IL(S)TEM areas.

In addition to the morphology analyses, EDS studies were also conducted in ILSTEM to track changes in the chemical composition of the nanocatalyst. Figure 5d shows the evolution of the oxygen content normalized by the sulfur in several IL(S)TEM areas (Normalized O at. % = 100(at. % O/(at. % O + at. % S)). To avoid the contribution of the oxygen content of the carbon layer of the TEM grid, the quantifications were performed in nanoflowers exposed to the vacuum of a hole in the TEM grid. The results show that the oxygen content dropped in most of nanoflowers analyzed, with an average decrease of 6 at. %. This is also observed when in the EDS maps, which show a lower oxygen content with respect to sulfur despite maintaining the morphology of the nanoflower assembly (Figure 5a, b). The initial oxygen content on the fresh material is related to the molybdenum precursor and the temperature used during the hydrothermal synthesis. Previous reports demonstrated that below 220 °C, this synthesis yields MoS_2_ with a percentage of Mo–O bonds stemming from unreacted molybdenum precursor.^38,39^ Such oxygen leads to an enhanced HER performance due to improved conductivity derived from a narrower band gap.^38^ Our IL(S)TEM investigation indicates that the reducing conditions at which the Re_0.2_Mo_0.8_S_2_ nanoflowers were subjected to evolve hydrogen lead to the reduction of this oxygen content. However, in the span of 4000 cycles, this decrease did not affect the HER activity of the Re_0.2_Mo_0.8_S_2_ nanocatalyst, which is mainly boosted by the rhenium content. As such, the stability of the Re is crucial for the long-term maintenance of the catalytic performance.^34,100^ Thus, the normalized Re at.% (normalized Re at. % = 100(Re at. %/(Re at. % + Mo at. %)) was also compared in IL(S)TEM by EDS. Contrary to the oxygen content, the Re content was maintained stable in all areas of analysis (Figure 5c, e) with an average of 21 at. % Re at cycles 0 and 4000, which explains the excellent stability of the Re_0.2_Mo_0.8_S_2_ activity for HER shown in Figure 3.

Overall, the IL(S)TEM results of the Re_0.2_Mo_0.8_S_2_ indicate high electrochemical stability and confirm that the tweezers method of electrical connection of the TEM grid in ILSTEM setup is suitable for the analysis of HER nanocatalysts, opening the door for a simple to implement, reliable, and reproducible method for expanding the potential of IL(S)TEM on gas evolving systems, which can contribute on the development of the next generation of electrocatalysts of such reactions.

## Conclusions

Different IL(S)TEM set-ups, namely the use of glassy carbon rod and Teflon cap, metallic wires, and metallic tweezers, were tested and compared for the study of HER nanocatalysts. The use of metallic tweezers proved to be the only easy and reliable approach to perform IL(S)TEM measurements for gas evolving reactions. With this approach, the stability of Re_0.2_Mo_0.8_S_2_ nanocatalysts was successfully analyzed by means of 4000 potential cycles until −0.25 V_RHE_, which are realistic and common conditions for the study of the stability of HER catalysts. The IL(S)TEM microscopy results confirmed excellent retention of the layered nanoflower morphology of the nanocatalyst, and the Re and Mo stoichiometric ratio was maintained stable. Nevertheless, the presence of oxygen stemming from the synthesis precursors, which has been related to an enhanced performance on MoS_2_-based nanocatalysts, was observed to decrease as a result of the electrochemical test. The approach to achieve reliable electrical contact can be directly applied to stability studies of other nanocatalysts and gas evolving reactions, expanding the potential of the IL(S)TEM to other areas of research where the stability of the catalysts is still the bottleneck.

## Experimental Section

### Materials

Sulfuric acid (H_2_SO_4_, Suprapur, Sigma-Aldrich) and deionized water (0.055 S/cm^2^) were used as electrolytes for electrochemical measurements. Ammonium heptamolybdate tetrahydrate, ((NH_4_)_6_Mo_7_O_24_·4H_2_O, 99.98%, ammonium perrhenate (NH_4_ReO_4_, > 99%, Merck), Sigma-Aldrich), thiourea (SC(NH_2_)_2_, > 99.0%, Sigma-Aldrich) and ethanol (EtOH, > 99.8, Carl Roth) were used for the synthesis of Re_0.2_Mo_0.8_S_2_ nanoflowers. Nafion (5 wt % in a mixture of alcohols and water, Sigma-Aldrich) and isopropanol (iProp, 99.9%, Schmitz) were used to prepare an ink to drop cast on the TEM grids. Hydrophilic carbon paper (CP, HCP030N, Hesen) substrates were used to synthesized Re_0.2_Mo_0.8_S_2_/CP electrodes.

### Synthesis

Re_0.2_Mo_0.8_S_2_ nanoflowers were synthesized with a hydrothermal approach.^40^ Ten milliliters of a solution containing 0.99 g of (NH_4_)_6_Mo_7_O_24_·4H_2_O, 2.28 g of SC(NH_2_)_2_ and 0.38 g of NH_4_ReO_4_ was heated for 20 h at 200 °C inside an autoclave. After cooling to room temperature, the black product was cleaned by centrifugation-redispersation cycles in water and ethanol. Finally, the suspension was dried at 110 °C, and the powder was ground in an agatha mortar.

To prepare Re_0.2_Mo_0.8_S_2_/CP electrodes the hydrothermal synthesis was modified by diluting 2700 times the concentration of the precursor solution and two carbon paper substrates of a size of 2 cm × 1 cm were included in the autoclave for heat treatment, with an area of 1 cm^2^ covered with Teflon tape. No nanoflowers grew on such a covered corner of the electrode, which was later used for making the electric connection with the potentiostat.

### Electrochemical Measurements

The IL(S)TEM electrochemical measurements were performed in a three-electrode setup with a Gamry Reference600 potentiostat using a reference hydrogen electrode (RHE, Gaskatel) as reference and a glassy carbon rod (6/60 mm, redoxme) as counter electrode. Holey carbon gold TEM finder grids (Plano) were used as working electrode. The working electrode connection was secured either with a glassy carbon electrode (3 mm electrode size, BioLogic), an Au wire (0.6 mm diameter, > 99.99%, redoxme), a Ti wire (0.5 mm, 99.99%, Thermofisher), reverted Au tweezers (Plano), or reverted Ti tweezers (Plano). The active surface area in contact with the electrolyte was limited using Teflon tape to wrap the Au wire and Au/Ti tweezers in order to control the background current and effectively estimate the surface of the working electrode for normalization of the current. The current density was calculated by dividing the current by the geometric active area (noncovered with Teflon) submerged in the electrolyte. All measurements were performed in 0.5 M H_2_SO_4_ degassed with Ar while stirring. Ohmic drop correction was applied to all measurements before plotting.

The electrochemical measurements on the Re_0.2_Mo_0.8_S_2_/CP electrode were conducted on an H-cell separated with a Nafion membrane. The reference electrode was an RHE and a GC rod was used as counter, while the electrolyte (0.5 M H_2_SO_4_) was degassed with Ar and continuously stirred. 4000 CVs were conducted at a scan rate of 100 mV/s from 0 to −0.25 V_RHE_, and every 1000 cycles a slow CV at a scan rate of 1 mV/s was acquired. The potentiostat used was a Metrohm PGSTAT-204. Ohmic drop correction was applied to all measurements before plotting to ensure fair comparison of the different electric contact methodologies.

### Electron Microscopy Measurements

To prepare the TEM grids, an ink containing 5 mg of Re_0.2_Mo_0.8_S_2_ catalyst powder and 10 μL of a Nafion solution in 2 mL of isopropanol was prepared from which 30 μL was drop cast.

The STEM measurements were conducted at a 300 kV acceleration voltage in a Titan Themis 60–300 from Thermofisher equipped with a probe aberration corrector. The EDS was performed using the Bruker Super X-EDS detector of the instrument. The SEM measurements were conducted with a ZEISS Gemini microscope with an in-lens secondary electron detector.

## Supporting Information Available

Additional electrochemical and structural characterizations (PDF)
