# Supplementary material for: Expanding the Potential of Identical Location Scanning Transmission Electron Microscopy for Gas Evolving Reactions: Stability of Rhenium Molybdenum Disulfide Nanocatalysts for Hydrogen Evolution Reaction
Source: ACS Appl Mater Interfaces. 2023 Sep 29;15(40):46895–901. doi: 10.1021/acsami.3c09188 (PMC10571005; doi:10.1021/acsami.3c09188)
Supplement: Supplementary file 1 — am3c09188_si_001.pdf [file am3c09188_si_001.pdf]

Supplementary Figures

Expanding the potential of identical location scanning transmission electron microscopy for gas evolving reactions: stability of rhenium molybdenum disulfide nanocatalysts for hydrogen evolution reaction

Miquel Vega-Paredes‡, Christina Scheu, Raquel Aymerich-Armengol*‡*

Max-Planck-Institut für Eisenforschung GmbH, Max-Planck-Str. 1, 40237 Düsseldorf, Germany

*C. Scheu: c.scheu@mpie.de

*R. Aymerich-Armengol: r.aymerich@mpie.de

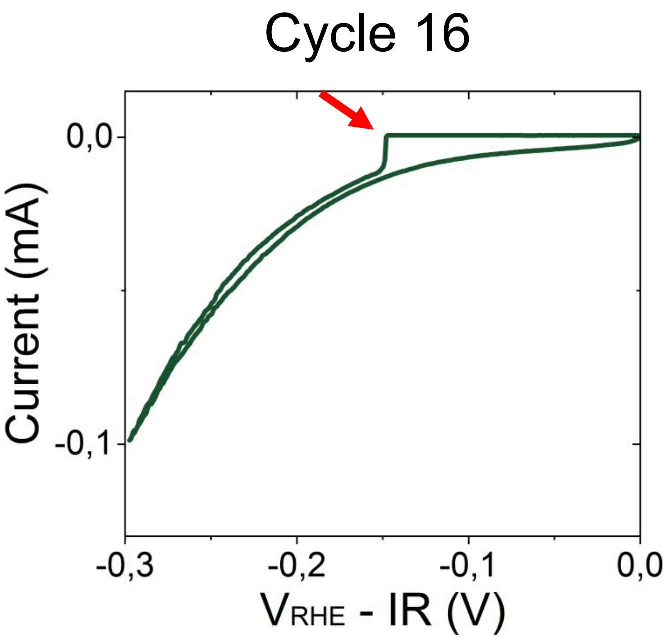

Figure S1. Loss of electrical contact in the 16th CV when cycling the sample using a glassy carbon rod electrode and Teflon cap for electrical contact and cycling from 0 to -0.3 V_{RHE} at 10mV/s.

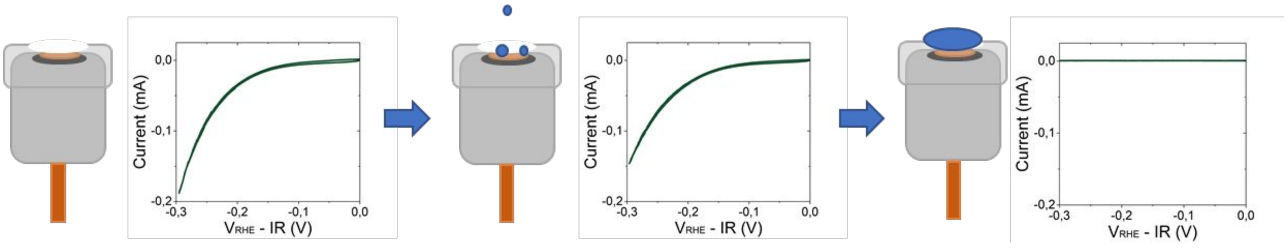

Figure S2. Mechanism of loss of electric contact due to gas evolution occurring when using the glassy carbon rod electrode and a Teflon cap.

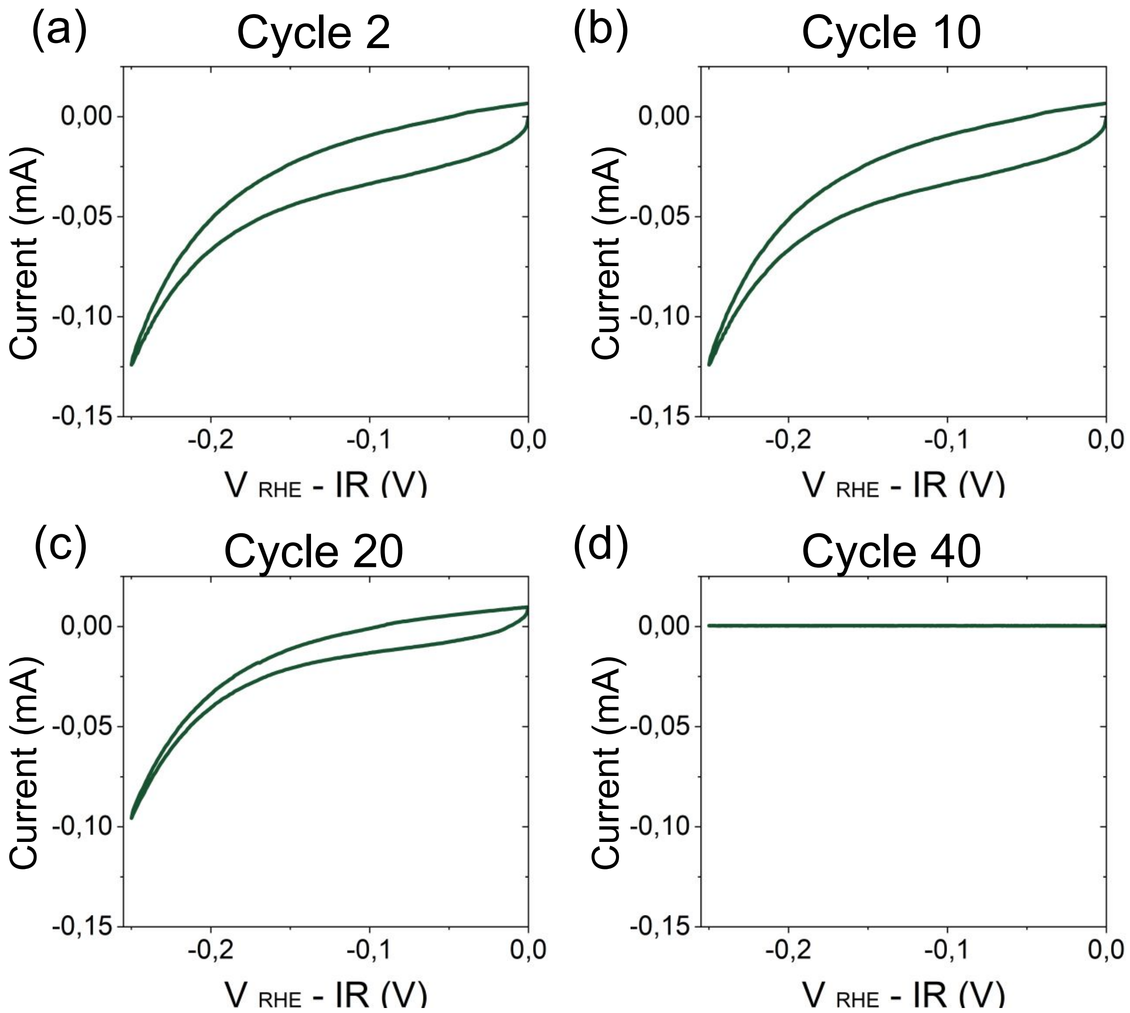

Figure S3. (a-d) Progressive loss of electrical contact when cycling the sample using the glassy carbon rod electrode with Teflon cap cycling from 0 to -0.25 V_{RHE} at 100mV/s. At the 40th cycle, there is no more measurable current due to loss of electric contact derived from bubbles.

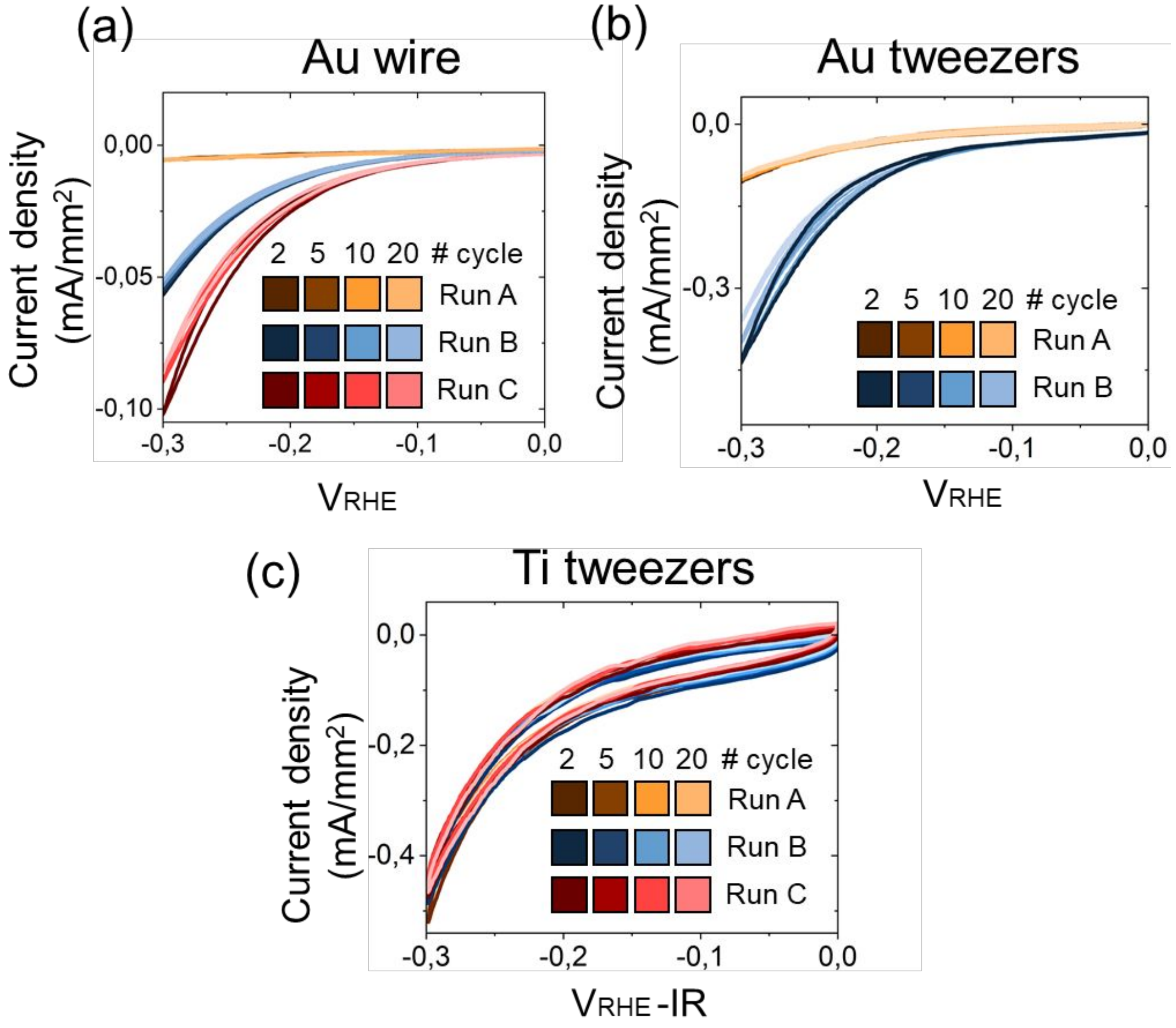

Figure S4. Different replicate runs of CVs performed on $Re_{0.2}Mo_{0.8}S_2$ nanocatalysts loaded on the Au TEM grid using (a) the Au wire, (b) the Au tweezers and (c) the Ti tweezers method. The electrochemical measurements were performed at a scan rate of 100 mV/s.

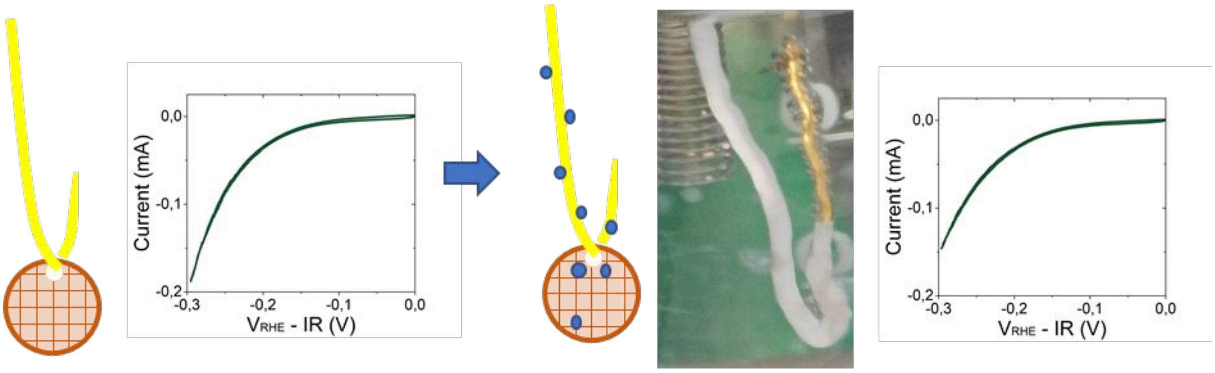

Figure S5. Growth of bubbles on the TEM grid and Au wire.

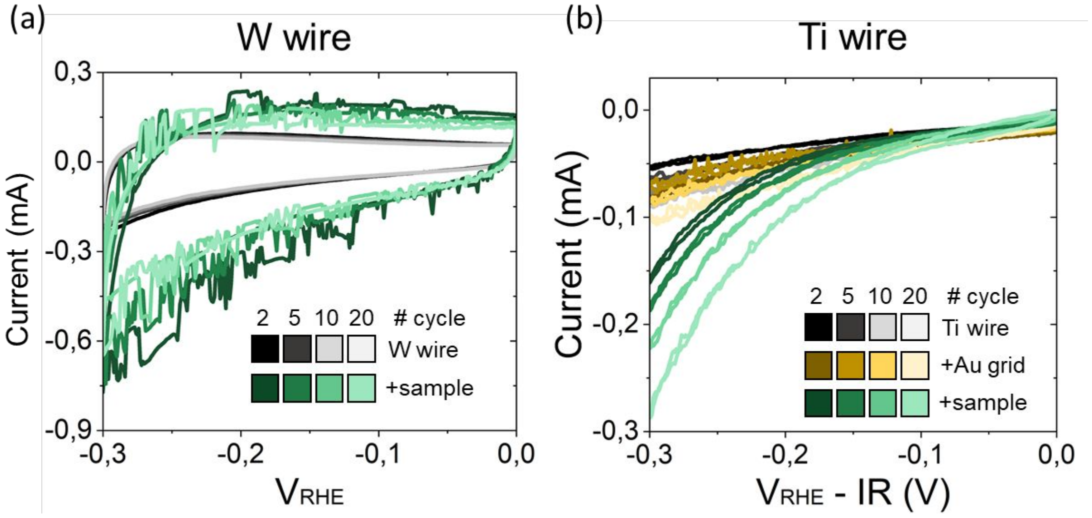

Figure S6. CV from 0 to -0.3V at 100 mV/s using a (a) W wire and (b) Ti wire. Notice that the Ti wire suffers from a clear surface activation process.

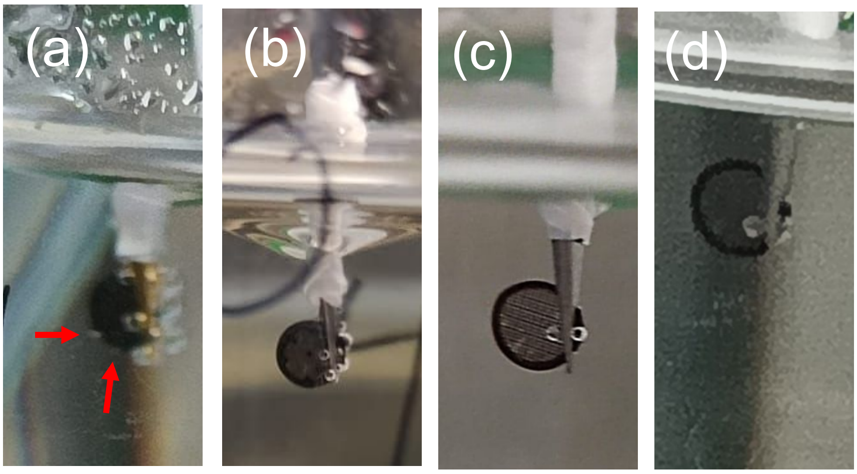

Figure S7. Gas evolution on the TEM grid using when cycling from 0 to -0.3 V vs. RHE using (a) Au tweezers, 1 mV/s of scan rate, cycle 20 (b) Ti tweezers, 1 mV/s of scan rate, cycle 20. Gas evolution on the TEM grid when cycling from 0 to -0.25 V vs. RHE using (c) Ti tweezers, 100 mV/s of scan rate, cycle 1000 (d) Ti tweezers, 100 mV/s of scan rate, cycle 4000. The TEM grid diameter is 3 mm. Notice that bubbling in the Au tweezers set-up is stronger and the bubbles are distributed all over the grid surface, while in the Ti tweezers approach only few bubbles stay exclusively at the tweezer-grid interface.

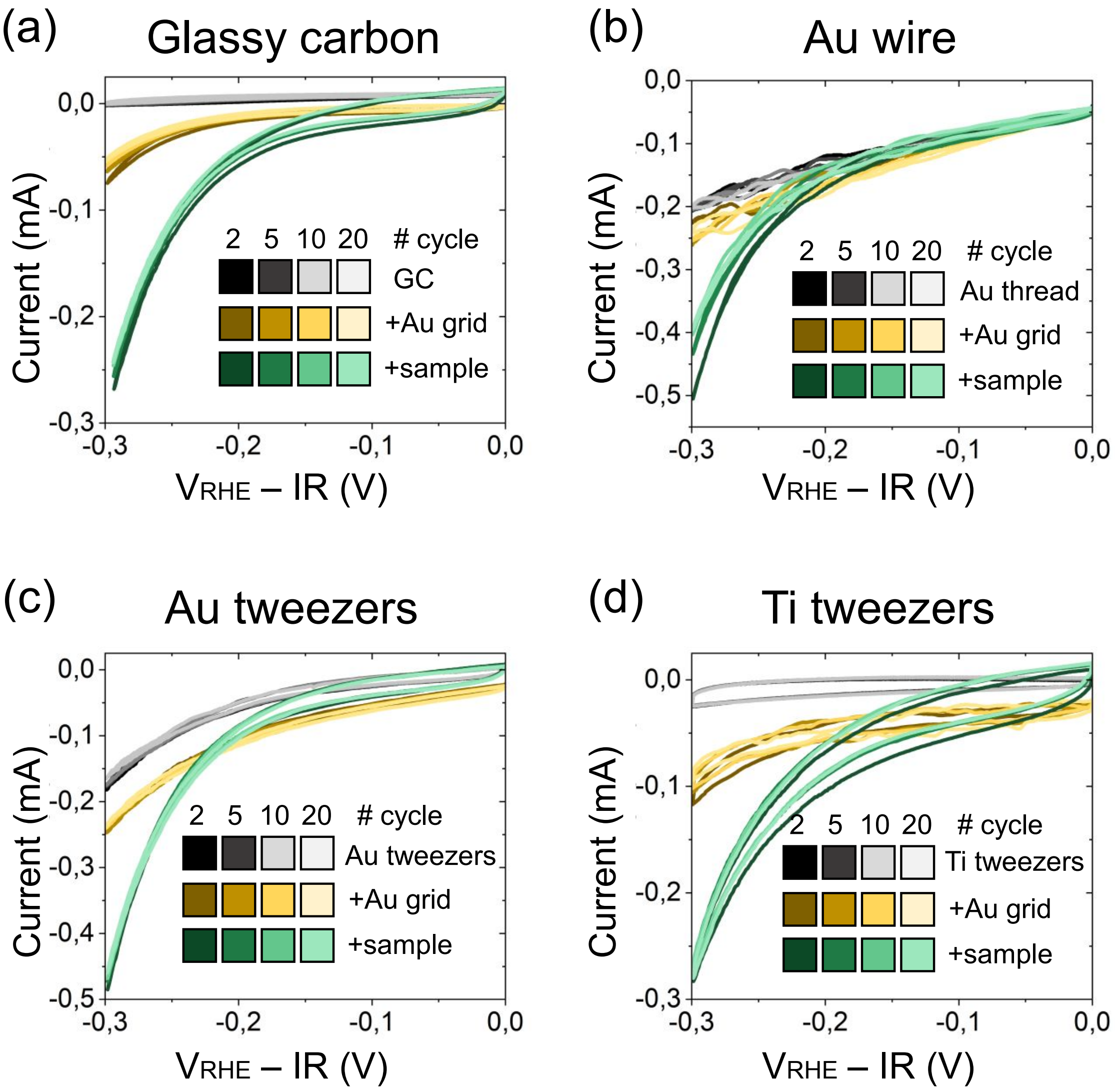

Figure S8. Comparison of the different methods of electrical connection of the TEM grids for IL(S)TEM at 100 mV/s, (a) Glassy carbon rod electrode, (b) Au wire, (c) Au tweezers and (d) Ti tweezers. The potential was cycled from 0 to -0.3 V vs. RHE for 20 cycles.

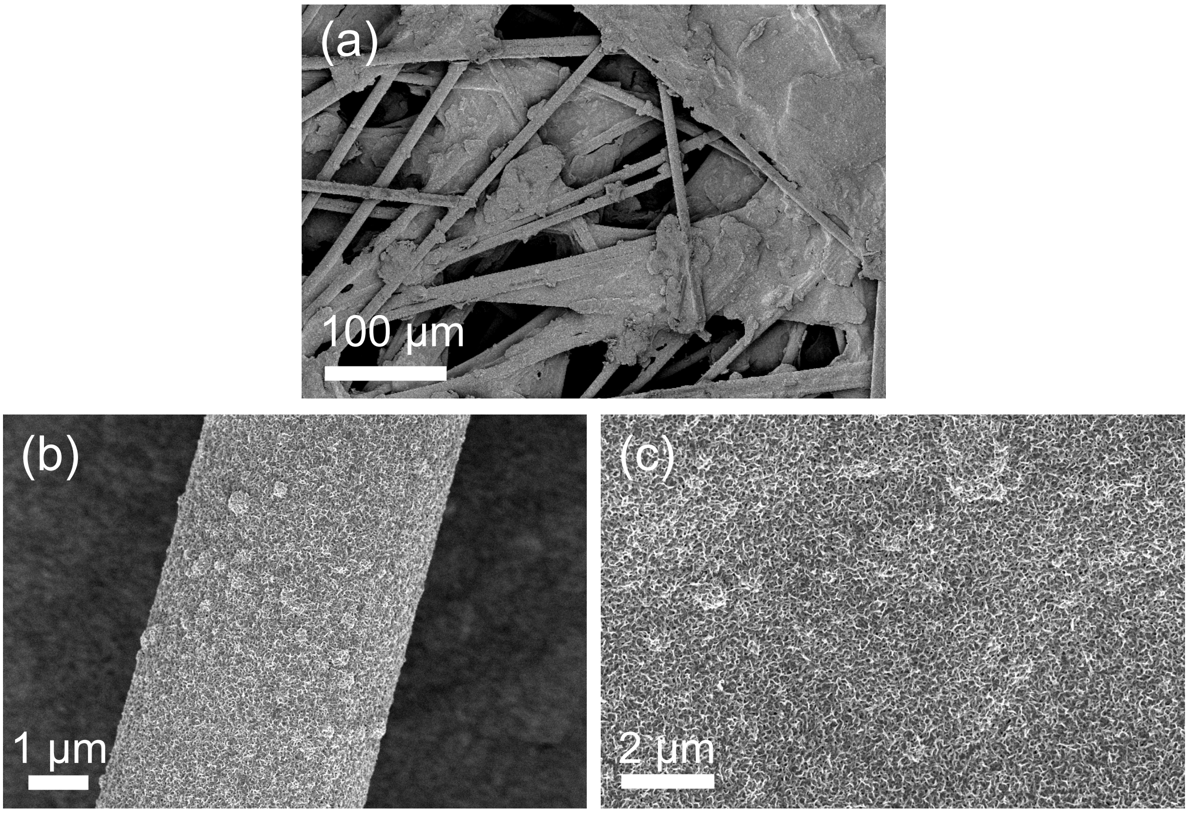

Figure S9. SEM micrographs showing the surface of $Re_{0.2}Mo_{0.8}S_2$/CP electrode. (a) Overview showing fibers and flat area. (b) Detail of a CP fiber covered with the $Re_{0.2}Mo_{0.8}S_2$ nanoflowers layer. (c) Detail of a CP flat area covered with the $Re_{0.2}Mo_{0.8}S_2$ nanoflowers.

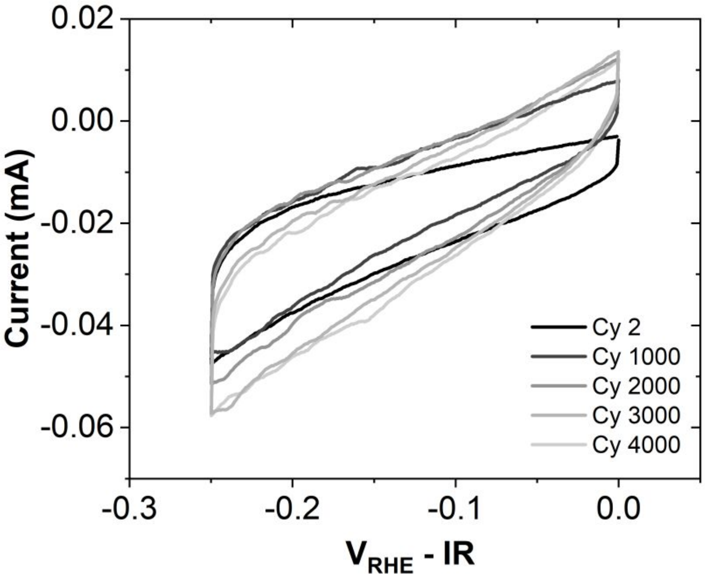

Figure S10. 4000 CV performed on the Ti tweezers from 0 to -0.25 V_{RHE} at a scan rate of 100 mV/s. Notice that there is an increasement of current that stabilizes after 3000 CV of value ~0.01 mA. An increasement of the same order of magnitude is observed in the current of the 4000 CV for the $Re_{0.2}Mo_{0.8}S_2$ nanocatalyst with Ti tweezers, confirming its origin in the tweezers themselves (**Figure 3b**). This increasement may be related to an activation of the Ti metal surface, which is passivated at air conditions. Despite the Ti being stable against corrosion at diluted sulfuric acid conditions, we hypothesize that such acid conditions summed to the reducing potential conditions of the HER regime may affect such passivation layer, leading to an increasement of current.

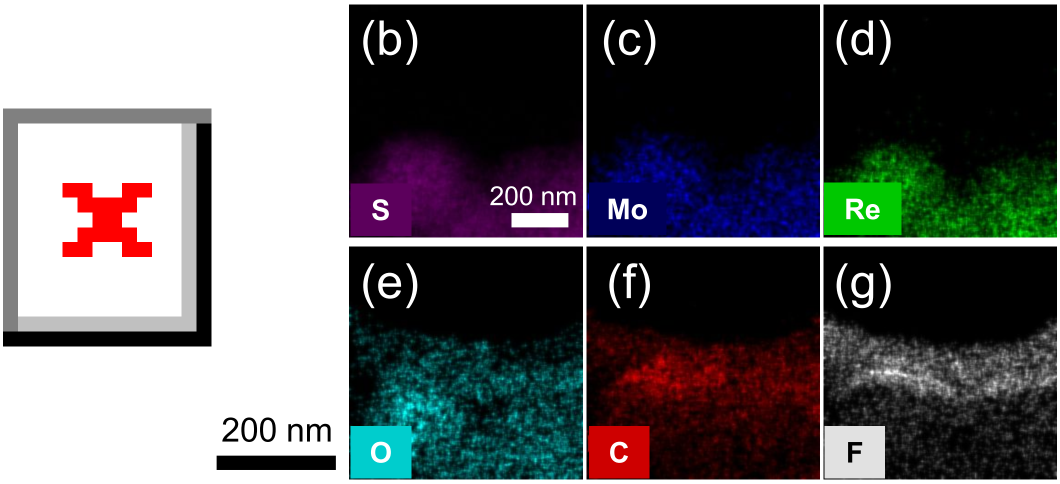

Figure S10. (a) HAADF-STEM image and corresponding EDS elemental maps (b-g) of an area with Nafion attached to the edge of the $Re_{0.2}Mo_{0.8}S_2$ nanocatalyst.
